# WDR-23 and SKN-1/Nrf2 Coordinate with the BLI-3 Dual Oxidase in Response to Iodide-Triggered Oxidative Stress

**DOI:** 10.1534/g3.118.200586

**Published:** 2018-08-30

**Authors:** Zhaofa Xu, Yiman Hu, Yajun Deng, Yutao Chen, Hanqi Hua, Siyu Huang, Qian Nie, Qian Pan, Dengke K. Ma, Long Ma

**Affiliations:** * Center for Medical Genetics, School of Life Sciences, Central South University, Changsha, Hunan 410078, China; † Cardiovascular Research Institute and Department of Physiology, UCSF School of Medicine, San Francisco, CA 94158-9001

**Keywords:** SKN-1, WDR-23, BLI-3 dual oxidase, reactive oxygen species, iodide

## Abstract

Animals utilize conserved mechanisms to regulate oxidative stress. The *C. elegans* SKN-1 protein is homologous to the vertebrate Nrf (NF-E2-related factor) family of cap ’n’ collar (CnC) transcription factors and functions as a core regulator of xenobiotic and oxidative stress responses. The WD40 repeat-containing protein WDR-23 is a key negative regulator of SKN-1 activity. We previously found that the oxidative stress induced by excess iodide can be relieved by loss of function in the BLI-3/TSP-15/DOXA-1 dual oxidase complex. To further understand the molecular mechanism of this process, we screened for new mutants that can survive in excess iodide and identified gain-of-function mutations in *skn-1* and loss-of-function mutations in *wdr-23*. The SKN-1C isoform functions in the hypodermis to affect animal’s response to excess iodide, while the SKN-1A isoform appears to play a minor role. *wdr-23(lf)* can interact with *bli-3* mutations in a manner different from *skn-1(gf)*. Transcriptome studies suggest that excess iodide causes developmental arrest largely independent of changes in gene expression, and *wdr-23(lf)* could affect the expression of a subset of genes by a mechanism different from SKN-1 activation. We propose that WDR-23 and SKN-1 coordinate with the BLI-3/TSP-15/DOXA-1 dual oxidase complex in response to iodide-triggered oxidative stress.

Animals utilize similar pathways in response to environmental or endogenous challenges from pathogenic, xenobiotic and oxidative stress (Kensler *et al.* 2007; Shore and Ruvkun 2013; Blackwell *et al.* 2015). At the core of the response is the conserved NRF2/Keap1 pathway in mammals or the SKN-1/WDR-23 pathway in *C. elegans* (Itoh *et al.* 1997; Itoh *et al.* 1999; An and Blackwell 2003; Kensler *et al.* 2007; Choe *et al.* 2009; Blackwell *et al.* 2015). When mammalian cells are under stress, Keap1 would dissociate from Nrf2, leading to the nuclear localization of Nrf2 and activation of gene expression that antagonizes the stress. In *C. elegans*, WDR-23 and SKN-1 appear to play similar roles as those of Keap1 and Nrf2 (Choe *et al.* 2009; Blackwell *et al.* 2015). The Nrf2/Keap1 pathway is also implicated in numerous human diseases, including neurodegeneration (Johnson and Johnson 2015), inflammation (Ahmed *et al.* 2017), cancer (Taguchi and Yamamoto 2017) and cardiovascular diseases (Barancik *et al.* 2016).

SKN-1 was initially identified as a maternally provided bZIP transcription factor unequally distributed in early embryos to specify the fate of pharyngeal and intestinal cells (Bowerman *et al.* 1992; Bowerman *et al.* 1993; Blackwell *et al.* 1994). SKN-1 is homologous to the vertebrate CNC-group proteins (NF-E2-related factors Nrf1 and Nrf2) with a highly conserved 14-amino-acid transactivator element (“DIDLID”) (Walker *et al.* 2000) and has a conserved function in regulating oxidative and xenobiotic stress responses by activating the phase II detoxification enzymes (An and Blackwell 2003). In embryos, SKN-1 is expressed in intestine and hypodermis, while in larvae and adults it appears to be highly expressed in the ASI neurons and weakly in intestinal cytoplasm and nuclei (An and Blackwell 2003). Oxidative or heat stress could significantly elevate the level of a SKN-1::GFP fusion protein in intestinal nuclei and enhance the expression of the phase II gene *gcs-1* (An and Blackwell 2003).

SKN-1 is a key regulator of the homeostasis of multiple cellular processes. It is required for lipid homeostasis (Steinbaugh *et al.* 2015), the expression of extracellular collagens for lifespan extension as a consequence of reduced Insulin/IGF-1 *INS-18* signaling (Ewald *et al.* 2015) and the stress response to cuticle damage (Dodd *et al.* 2018). Mitochondrial proline catabolism can activate SKN-1 to affect lifespan (Zarse *et al.* 2012) or innate immunity (Tang and Pang 2016), and a mitochondria-associated gain-of-function SKN-1 could mediate a conserved starvation response even with *ad lib* access to food (Paek *et al.* 2012). SKN-1 is also involved in mitophagy (Palikaras *et al.* 2015) and can be activated by the IRE protein sulfenylated by ER- or mitochondria-derived ROS (Hourihan *et al.* 2016). The crosstalk between SKN-1 and mitochondria appears to be conserved across species (Itoh *et al.* 2015). SKN-1 activity is regulated by multiple signals (An *et al.* 2005; Inoue *et al.* 2005; Kell *et al.* 2007; Tullet *et al.* 2008; Wang *et al.* 2010; Li *et al.* 2011; Robida-Stubbs *et al.* 2012; Glover-Cutter *et al.* 2013; Ruf *et al.* 2013; Ewald *et al.* 2015).

WDR-23 is a conserved WD40 repeat-containing protein that interacts with the CUL4-DDB1 ubiquitin ligase to promote ubiquitin proteasome system-mediated degradation of SKN-1 in *C. elegans* (Choe *et al.* 2009). *wdr-23* loss-of-function mutations can lead to constitutive expression of phase II genes, which is similar to the effect of SKN-1 activation (Hasegawa and Miwa 2010). In mammals, a similar WDR23-DDB1-CUL4-dependent mechanism can repress Nrf2 activity independent of the canonical KEAP1-CUL3 pathway, suggesting that WDR-23-dependent regulation of SKN-1 is conserved (Lo *et al.* 2017).

Several lines of evidence suggest that SKN-1 and the *C. elegans* NADPH dual oxidase BLI-3 DUOX1 might act together in response to stress. Manganese (Mn)-induced toxicity requires the activity of BLI-3, while SKN-1 can protect against Mn toxicity (Benedetto *et al.* 2010). Bacterial or fungal pathogens can trigger BLI-3-dependent ROS generation (Chavez *et al.* 2009; Zou *et al.* 2013), which can activate SKN-1 target gene expression (Hoeven *et al.* 2011; Papp *et al.* 2012; Van Der Hoeven *et al.* 2012). Loss of the mammalian mediator of ErbB2-driven cell motility, MEMO-1, could lead to enhanced production of ROS by BLI-3, which stimulates SKN-1 to promote stress resistance and longevity (Ewald *et al.* 2017). Similarly, a redox co-factor, pyrroloquinoline quinone could activate BLI-3 to produce H_2_O_2_ at plasma membrane, the effect of which is transduced by SKN-1, JUN-1 and DAF-16 for lifespan extension (Sasakura *et al.* 2017). These findings suggest that the BLI-3 dual oxidase activity and the SKN-1 activity are probably coordinated to respond to oxidative stress and maintain ROS homeostasis.

Iodine is a micronutrient essential for life and a key ingredient for the synthesis of thyroid hormones. Insufficient intake of iodide can lead to thyroid hormone deficiency and cause severe hypothyroidism and mental retardation (Nussey and Whitehead 2001). However excess iodide intake has been implicated in autoimmune thyroiditis (Bagchi *et al.* 1985; Rose *et al.* 1997; Rose *et al.* 1999; Teng *et al.* 2006), hyperthyroidism (Nussey and Whitehead 2001), hypothyroidism (Rose *et al.* 1999; Teng *et al.* 2006) and thyroid cancers (Lind *et al.* 1998; Guan *et al.* 2009; Blomberg *et al.* 2012; Dong *et al.* 2013). The molecular mechanism underlying the pathogenic effects of excess iodide is unclear.

We recently used *C. elegans* as a model to analyze the xenobiotic effect of excess iodide. We found that excess iodide could cause larval arrest, cuticle shedding defects and premature intestinal autofluorescence, phenotypes that can be reversed when animals were moved to normal growth media (Xu *et al.* 2014). A screen for mutants that can survive in excess iodide isolated loss-of-function (lf) mutations in *bli-3* and *tsp-15* (Xu *et al.* 2014), in which *tsp-15* encodes a tetraspanin protein required for BLI-3 activity (Moribe *et al.* 2004; Moribe *et al.* 2012). We found that the BLI-3/TSP-15/DOXA-1 dual oxidase complex is required for the xenobiotic effects of excess iodide (Xu *et al.* 2014), which might involve iodide-induced excessive generation of ROS (Xu *et al.* 2014).

In this study, we report the identification of novel gain-of-function mutations in *skn-1* and loss-of-function mutations in *wdr-23* and *bli-3/tsp-15/doxa-1* complex and how these genes interact to affect *C. elegans* cuticle integrity and survival in excess iodide. Besides verifying the known interaction between WDR-23 and SKN-1 in stress responses, we found that *wdr-23* can interact with *bli-3* and affect gene expression by a mechanism different from SKN-1 activation.

## Materials and Methods

### Strains

See Supplementary Materials and Methods.

### C. elegans survival in excess iodide

The survival assay was performed as described (Xu *et al.* 2014). In short, five young adults were grown on *E. coli* OP50-seeded NGM plates with different concentrations of NaI (5 mM, 10 mM or 50 mM). F_1_ progeny were observed for growth and survival until day 8. For transient transgenic experiments, P_0_ adult animals injected with transgenes were transferred to OP50-seeded NGM plates with 5 mM NaI and transgene-positive F_1_ progeny were examined for growth to adults.

### Genetic screens and mapping of mutations

See Supplementary Materials and Methods.

### Hoechst 33258 staining

Hoechst 33258 staining was performed as described (Moribe *et al.* 2004; Xu *et al.* 2014) with minor modifications. Synchronized animals (24 hr post mid-L4) were washed off plates and incubated at 20° with gentle shaking for 15 min with 1 μg/ml Hoechst 33258 (Sigma) diluted in M9. After staining, animals were washed three times with M9 and observed under a Leica DM5000B fluorescence microscope.

### RNA interference

L4 animals were fed HT115 (DE3) bacteria expressing dsRNAs on NGM plates with 1 mM IPTG, 0.1 mg/ml Ampicillin (Timmons *et al.* 2001) with or without 5 mM NaI for 8 days. The progeny were examined under dissecting microscope for survival. The RNAi feeding bacterial strains for *wdr-23* and *skn-1* were obtained from a whole-genome RNAi library (Kamath *et al.* 2003), and the inserts were verified by sequencing. The *doxa-1* RNAi feeding bacterial strain was described previously (Xu *et al.* 2014).

### Plasmids

See Supplementary Materials and Methods.

### Transgene experiments

See Supplementary Materials and Methods.

### qRT-PCR

Synchronized animals at the L1 larval stage were allowed to recover on OP50-seeded NGM plates with or without 5 mM NaI for 8 hr and subsequently washed three times with H_2_O. RNA was extracted using TRIzol (Invitrogen) and chloroform-isopropanol purification and treated with DNase I (NEB). RNA concentration and quality were measured with a NanoDrop 1000 spectrophotometer (Thermo Fisher). cDNAs were prepared using the Maxima First Strand cDNA Synthesis Kit for qRT-PCR (Thermo Fisher). mRNA levels were quantified from three biological replicates using Maxima SYBR Green (Thermo Fisher) fluorescence on a LightCycler 96 Instrument (Roche). After a pre-incubation step (95° for 10 min), two-step amplification was performed using 40 cycles of denaturation (95° for 15 s) and annealing (60° for 45 s). Target gene expression levels were normalized to that of the reference gene *tba-1*. Primers for constructs and qRT-PCR experiments are listed in Table S7.

### Transcriptome analyses

See Supplementary Materials and Methods.

### Statistics

*P* values were determined by two-tailed unpaired Student’s *t*-test for comparisons between two samples and Bonferroni test with one-way ANOVA for comparisons of more than two samples.

*: *P* < 0.05; **: *P* < 0.01; ***: *P* < 0.001.

### Data Availability

Strains and plasmids are available upon request. The authors affirm that all data necessary for confirming the conclusions of the article are present within the article, figures, and tables. Gene expression data are available at GEO with the accession number: GSE117222. Supplemental material available at Figshare: https://doi.org/10.25387/g3.6983384.

## Results

### Loss-of-function mutations in wdr-23 and gain-of-function mutations in skn-1 can promote C. elegans survival in excess iodide

**Figure 1:**
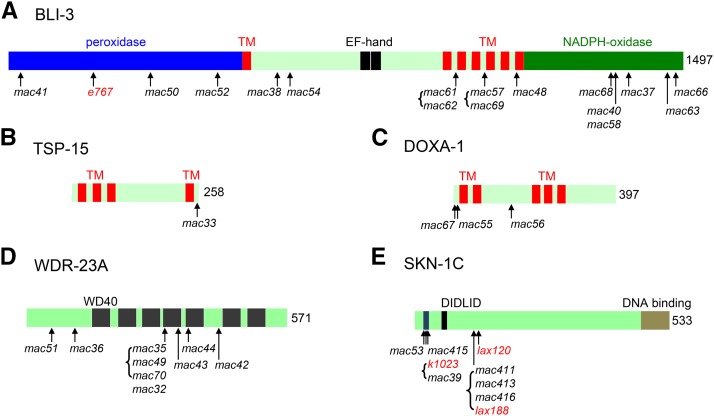
Protein domain structures and positions of mutations in BLI-3 (A), TSP-15 (B), DOXA-1 (C), WDR-23A (D) and SKN-1C (E). Protein domains are labeled. In (E), “DIDLID” is conserved between SKN-1C and mammalian Nrf2. TM, transmembrane domain. Brackets indicate that the enclosed mutations cause identical amino acid changes.

In a previous screen for mutants that can survive in excess iodide (5 mM NaI) (Xu *et al.* 2014) (Table S1, Screen 1), we isolated four lf mutations in *bli-3* (*mac37*, *mac38*, *mac40*, *mac41*) and one lf mutation in *tsp-15* (*mac33*) (Table S1, Table S2 and Figure 1). The lf nature of the *tsp-15(mac33)* mutation was further confirmed by transgene rescue experiments in this study (Table S3), showing that wild-type *tsp-15* transgenes nearly abolished the survival of *tsp-15(mac33lf)* mutants in excess iodide.

From Screen 1 (Table S1), we also isolated six mutations (*mac32*, *mac35*, *mac36*, *mac42*, *mac43 and mac44*) that form a distinct complementation group on Chr. I. SNP mapping and candidate gene analyses indicate that they affect *wdr-23* (Table S2 and Figure 1). *wdr-23* encodes a WD40 repeat-containing protein that functions with the CUL-4/DDB-1 ubiquitin ligase to negatively regulate the activity of SKN-1 (Choe *et al.* 2009). Both *wdr-23(mac32)* homozygous animals and animals fed RNAi targeting *wdr-23* can survive in excess iodide, while *wdr-23(mac32)* heterozygous animals failed to (Table 1). We could rescue the survival-promoting effect of the *wdr-23(mac32)* mutation using either *wdr-23a* or *wdr-23b* isoform transgenes (Table S3), suggesting that these mutations cause loss of function in *wdr-23*.

**Table 1:** The survival of different mutants and wild-type animals treated with RNAi in excess iodide

Genotype	Survival in 5 mM NaI
*WT*	No
*control RNAi*	No
***wdr-23(mac32)***	**Yes**
*wdr-23(mac32)/+*	No
***wdr-23(RNAi)***	**Yes**
***skn-1(mac39)***	**Yes**
***skn-1(mac39)/+***	**Yes**
***skn-1(lax120gf)***	**Yes**
***skn-1(lax120gf)/+***	**Yes**
*skn-1(zu135lf)*	No
*skn-1(RNAi)*	No
***doxa-1(mac55)***	**Yes**
*doxa-1(mac55)/+*	No
***doxa-1(RNAi)***	**Yes**

We mapped the last mutation (*mac39*) in Screen 1 to Chr. IV within a region containing *skn-1*. Knowing that WDR-23 is a negative regulator of SKN-1 (Choe *et al.* 2009), we took *skn-1* as a candidate and indeed identified a missense mutation that causes an R43C amino acid change (Table S1, Table S2 and Figure 1) on the SKN-1C isoform in *mac39* mutants.

Both *mac39* heterozygous and homozygous animals can survive in excess iodide, while the *skn-1(zu135lf)* (Bowerman *et al.* 1992) homozygous mutants and animals fed RNAi targeting *skn-1* failed to (Table 1). In addition, both heterozygous and homozygous animals of the previously identified *skn-1(lax120gf)* mutation (Paek *et al.* 2012) can survive in excess iodide (Table 1). Based on these findings, we propose that *mac39* causes a gain of function (gf) in *skn-1*.

### Additional screens isolated novel lf mutations in bli-3, doxa-1 and wdr-23 and gf mutations in skn-1

To identify more genes and mutations involved in animal’s response to excess iodide, we performed additional screens (Table S1) for mutants that can survive in 5 mM NaI potentially as homozygotes (Screen 2 for F_2_ mutants) or heterozygotes (Screen 3 for F_1_ mutants).

In total, 33 independent mutants were isolated. Genetic and sequence analyses identified 16 lf mutations in *bli-3* (*mac48*, *mac50*, *mac52*, *mac54*, *mac57*, *mac58*, *mac59*, *mac60*, *mac61*, *mac62*, *mac63*, *mac64*, *mac65*, *mac66*, *mac68* and *mac69*), three lf mutations in *wdr-23* (*mac49*, *mac51* and *mac70*), five gf mutations in *skn-1* (*mac53*, *mac411*, *mac413*, *mac415* and *mac416*) and three lf mutations in *doxa-1* (*mac55*, *mac56* and *mac67*). (Figure 1, Tables S1 and S2). We are currently investigating the genetic changes in six remaining isolates that can survive in excess iodide as heterozygotes (Table S1).

Among the nine *wdr-23* mutants, *mac35*, *mac49* and *mac70* cause an identical D312N amino acid change in the 4^th^ WD40 domain. The same mutation was previously described in the *xrep-1(k1011lf)* (*wdr-23*) mutant that exhibits constitutive expression of phase II enzymes (Hasegawa and Miwa 2010). *mac32* and *mac43* cause a D313N change and a G331R change in the 4^th^ WD40 domain, respectively, and *mac44* causes a G361R change in the 5^th^ WD40 domain (Table S2 and Figure 1). *mac36*, *mac42 and mac51* cause nonsense mutations at residues R130, W413 and Q80 (Figure 1 and Table S2), respectively.

All *skn-1* mutants can survive in excess iodide as heterozygotes or homozygotes. Using *skn-1c* as the reference isoform, the amino acid change (G39D) in *skn-1(mac53)* (Table S2 and Figure 1) is one amino acid away from the R41C change caused by *skn-1(k1023gf)* (Tang and Choe 2015) and three amino acids away from the R43C change caused by *skn-1(mac39gf)*, suggesting functional importance of a potential domain in SKN-1C that contains these amino acid residues. The *skn-1(mac415)* mutation causes the same R41C change in SKN-1C as that by *skn-1(k1023gf)* (Tang and Choe 2015). The *mac411*, *mac413*, *mac416* mutations cause an E147K amino acid change identical to the previously described *skn-1(lax188gf)* mutation (Paek *et al.* 2012). Therefore, we isolated new *skn-1* gf mutations as well as mutations that were previously described.

The new screens also isolated mutations in *doxa-1* (*mac55*, *mac56*, *mac67*) (Table S1). *doxa-1* encodes an ortholog of the mammalian dual oxidase maturation factor (Moribe *et al.* 2012). Both *mac55* homozygous animals (Table 1) and animals fed RNAi targeting *doxa-1* (Table 1) (Xu *et al.* 2014) can survive in excess iodide, while *mac55* heterozygous animals failed to (Table 1). The survival-promoting effect of *mac55* could be rescued by wild-type *doxa-1* transgenes (Table S3), suggesting that *mac55* and the other two mutations cause loss of function.

### SKN-1C functions in the hypodermis (epidermis) to promote animal survival in excess iodide

*skn-1* is predicted to express four isoforms, *skn-1a*, *skn-1b*, *skn-1c and skn-1d* (Blackwell *et al.* 2015), among which *skn-1c* has been extensively studied. In larvae and adults, *skn-1c* is apparently expressed in ASI neurons and weakly in intestine (An and Blackwell 2003). *skn-1c* functions in intestine to regulate a variety of biological processes (Blackwell *et al.* 2015). SKN-1C expression has also been observed in hypodermis using *skn-1c* transgenes (Wu *et al.* 2016) and in hypodermis, pharynx and body-wall muscles based on the expression of SKN-1C target genes (Hasegawa *et al.* 2008; Paek *et al.* 2012).

**Figure 2:**
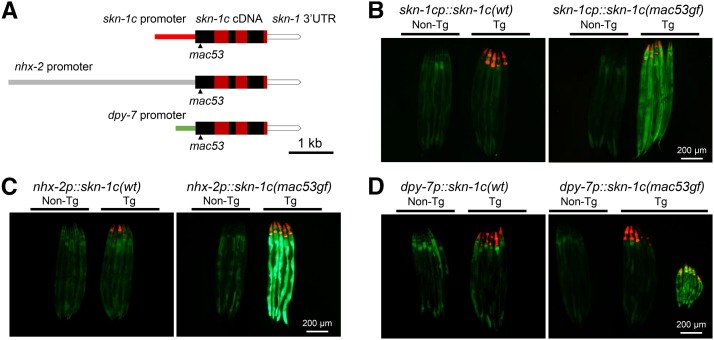
*skn-1c(mac53gf)* transgenes under control of *skn-1c* or *nhx-2* promoters can increase the *gst-4p*::*GFP* (*dvIs19*) reporter expression. (A) Structures of transgenes for phenocopying the survival-promoting effect of *skn-1c(gf)* mutations. The coding exons of *skn-1c* are delineated as alternating red and black boxes. (B, C, D) Left panels. *skn-1c(wt)* transgenes did not increase the expression of the *gst-4p*::*GFP* reporter. Non-transgenic (Non-Tg) animals are on left. Transgenic animals (Tg, red fluorescence in pharynxes expressed from the pCFJ90 co-injection marker) are on right. (B, C, D) Right panels, *skn-1c(mac53gf)* transgenes. Non-transgenic and transgenic animals are indicated on left and right, respectively. (D) Right panel, Tg: *dpy-7p*::*skn-1c(mac53gf*) transgenes did not increase the expression of the *gst-4p*::*GFP* reporter in most cases (animals on the left). Occasionally the transgene caused a strong Dpy phenotype with increased reporter expression in the intestine (animals on the right).

To identify the tissue(s) in which *skn-1(gf)* mutations promote the survival in excess iodide, we performed phenocopy experiments by introducing *skn-1c* transgenes to wild-type animals. Stable *skn-1c(wt)* cDNA transgenes under control of a *skn-1c* endogenous promoter (An and Blackwell 2003) (Fig. S1A) (Figure 2A), an intestine-specific *nhx-2* promoter (Nehrke 2003) (Figure 2A) or *ges-1* promoter (Egan *et al.* 1995), a hypodermis-specific *dpy-7* promoter (Gilleard *et al.* 1997) (Figure 2A) or a body-wall muscle-specific *myo-3* promoter (Okkema *et al.* 1993), all failed to promote the survival (Table S4).

We next examined whether the *skn-1c(mac53gf)* cDNA transgene could promote the survival in excess iodide. We established stable *skn-1c(mac53gf)* transgenic lines using the two intestine-specific promoters (*nhx-2p* and *ges-1p*) and the body-wall muscle-specific *myo-3* promoter. However, these transgenic animals could not survive in excess iodide (Table S4), suggesting that *skn-1(gf)* might not function in intestine or muscle to promote the survival.

Surprisingly, we failed to establish stable *skn-1c(mac53gf)* lines using the *skn-1c* promoter or the hypodermis-specific *dpy-7* promoter (Table S4), probably due to the toxicity of hypodermis-specific *skn-1c(gf)* overexpression. Such toxicity is only obvious in the F_2_ generation as we could generate abundant viable *skn-1(gf)* transgene-positive F_1_ animals (Table 2).

**Table 2:** Hypodermis-specific *skn-1c(gf)* expression can promote animal survival in excess iodide

Transient transgene	Experiment (No. injected WT P_0_)	No. survived Tg F_1_ adults (No. Tg F_1_)	Percentage
*skn-1cp*::*skn-1c(wt)* (*skn-1c* endogenous promoter)	1 (15)	0(28)	0.00%
	2 (15)	0(70)	0.00%
	3 (15)	0(34)	0.00%
***skn-1cp*::*skn-1c(mac53gf)* (*skn-1c* endogenous promoter)**	1 (20)	**37(129)**	**28.68%**
	2 (20)	**31(153)**	**20.26%**
	3 (20)	**52(181)**	**28.73%**
	4 (20)	**28(158)**	**17.72%**
*dpy-7p*::*skn-1c(wt)* (hypodermis promoter)	1 (15)	0(37)	0.00%
	2 (15)	0(21)	0.00%
	3 (15)	0(26)	0.00%
***dpy-7p*::*skn-1c(mac53gf)* (hypodermis promoter)**	1 (15)	**31(46)**	**67.39%**
	2 (15)	**23(63)**	**36.51%**
	3 (15)	**49(74)**	**66.22%**

To overcome the toxicity of stable *skn-1c(gf)* transgenes under control of the *skn-1c* or the *dpy-7* promoter, we examined the survival of *skn-1c(mac53gf)* transgene-positive (based on co-injection marker expression) F_1_ progeny in excess iodide. Here, we found that numerous transgene-positive F_1_ animals could grow into adults in excess iodide (Table 2) and the *dpy-7* promoter appears to be more robust than the *skn-1c* promoter. Therefore, *skn-1c(gf)* can function in the hypodermis to promote the survival.

Since we isolated lf mutations in the *bli-3/tsp-15/doxa-1* complex in the same screens, we tested whether these genes function in the hypodermis as well. Indeed, stable *tsp-15* cDNA transgenes under control of the *dpy-7* promoter could strongly rescue the survival-promoting effect of the *tsp-15(mac33lf)* mutation (Table S3), suggesting that the *bli-3/tsp-15/doxa-1* complex also functions in the hypodermis to affect the survival in excess iodide.

### skn-1c(mac53gf) transgenes can activate the expression of SKN-1C target gene *gst-4*

To test whether the failure of intestine-specific *skn-1c(gf)* transgene expression in promoting the survival might be caused by a lack of activated SKN-1C target gene expression, we introduced *skn-1c(mac53gf)* transgenes to the *dvIs19* transgenic animals (Link and Johnson 2002). The *dvIs19* transgene expresses GFP under control of the *gst-4* promoter and is used as a reliable reporter for SKN-1C activation.

We found that *skn-1c(mac53gf)* transgenes, under control of either *skn-1c* promoter or the intestine-specific *nhx-2* promoter, can significantly increase GFP expression in the intestines of transgene-positive F_1_ progeny (Figure 2B and 2C, right panels), while *skn-1c(wt)* transgenes have no obvious effect (Figure 2B and 2C, left panels). The *nhx-2* promoter appears to cause a more robust GFP expression than the *skn-1c* promoter does.

Under control of the *dpy-7* promoter, the *skn-1c(mac53gf)* transgene resulted in two distinct groups of F_1_ transgenic progeny. In most cases, the transgenic animals have normal size with normal intestinal GFP expression and can grow into adults in excess iodide (Figure 2D, right panel, animals on the left under Tg). Occasionally, we found transgenic animals with a strong Dpy phenotype and an apparent increase in intestinal GFP expression that failed to grow in excess iodide (Figure 2D, right panel, animals on the right under Tg). The mechanism underlying these distinct phenotypes might be related to the difference in levels, temporal stages or leakiness of the transgene expression. These results together suggest that intestinal activation of SKN-1C is not sufficient for animal survival in excess iodide.

### The SKN-1A isoform can weakly promote animal survival in excess iodide

The *skn-1(gf)* mutations that we isolated affect SKN-1A and SKN-1C isoforms, but not SKN-1B or SKN-1D isoforms (Fig. S1). SKN-1A is expressed in most tissues (An and Blackwell 2003; Bishop and Guarente 2007; Staab *et al.* 2014) and associated with ER to mediate transcriptional activation of proteasome subunit genes upon proteasome disruption (Lehrbach and Ruvkun 2016). Oxidative and ER stress can increase *skn-1a* expression (Glover-Cutter *et al.* 2013).

To examine whether *skn-1a* plays a role in animal’s response to excess iodide, we generated transgenic lines (Table S4) with *skn-1a(wt)* or *skn-1a(mac53gf)* cDNA under control of the *skn-1a* (Staab *et al.* 2014) or *skn-1c* promoter (An and Blackwell 2003) (Fig. S1A). All transgenic lines failed to survive in excess iodide (Table S4). However, we consistently found escapers in the *skn-1a(mac53gf)* lines controlled by the *skn-1a* promoter (Table S4). To verify this finding, we examined the survival of F_1_ *skn-1a* transgenic animals in excess iodide (Table S5). The results suggest that both *skn-1a(wt)* and *skn-1a(gf)* transgenes under control of either *skn-1c* or *skn-1a* promoter could weakly promote the survival.

To examine whether *skn-1a* transgenes might affect *skn-1c* target gene expression, we introduced these transgenes to the *dvIs19* animals. It appears that the *skn-1a(wt)* or *skn-1a(gf)* transgenes under control of the *skn-1a* promoter could weakly activate the GFP expression (Fig. S1B, two left panels), while these transgenes under control of the *skn-1c* promoter failed to do so (Fig. S1B, two right panels). These results suggest that both *skn-1a(wt)* and *skn-1a(gf)* are capable of weakly activating SKN-1C target gene expression. Furthermore, *skn-1a(wt)* might carry an activity similar to *skn-1a(gf)* in promoting animal survival in excess iodide. The underlying mechanism remains to be understood.

### skn-1 is required for the survival of bli-3, tsp-15, doxa-1 and wdr-23 lf mutants in excess iodide

Since *skn-1(zu135lf)* and *skn-1(RNAi)* animals could not survive to adults in excess iodide (Table 1), we examined whether *skn-1* is epistatic to *bli-3*, *tsp-15*, *doxa-1* or *wdr-23*. We generated double mutants carrying the *skn-1(zu135lf)* mutation and one of two or more independently isolated mutations in these other genes. Except for *bli-3(e767lf)*; *skn-1(zu135lf)* double mutants, which were too sick for survival test, all other double mutants grew similarly as *skn-1(zu135lf)* single mutants under normal condition but failed to survive to adults in excess iodide (Table 3). Therefore, *skn-1* is required for mutations in the other four genes to promote animal survival in excess iodide.

**Table 3:** *skn-1* is required for the survival of *bli-3*, *tsp-15*, *doxa-1* and *wdr-23* lf mutants in excess iodide

Genotype		Growth in 5 mM NaI
*skn-1(lf)*	*zu135/nT1[qIs51]* (heterozygous)	Larval arrest
	*zu135* (homozygous)	Larval arrest
*bli-3(lf)*; *skn-1(lf)*	*e767*; *zu135/nT1[qIs51]*	ND
	*e767*; *zu135*	ND
	*mac52*; *zu135/nT1[qIs51]*	Adult
	*mac52*; *zu135*	Larval arrest
	*mac40*; *zu135/nT1[qIs51]*	Adult
	*mac40*; *zu135*	Larval arrest
	*mac66*; *zu135/nT1[qIs51]*	Adult
	*mac66*; *zu135*	Larval arrest
*tsp-15(lf)*; *skn-1(lf)*	*sv15*; *zu135/nT1[qIs51]*	Adult
	*sv15*; *zu135*	Larval arrest
	*mac33*; *zu135/nT1[qIs51]*	Adult
	*mac33*; *zu135*	Larval arrest
*doxa-1(lf)*; *skn-1(lf)*	*mac55*; *zu135/nT1[qIs51]*	Adult
	*mac55*; *zu135*	Larval arrest
	*mac67*; *zu135/nT1[qIs51]*	Adult
	*mac67*; *zu135*	Larval arrest
*wdr-23(lf)*; *skn-1(lf)*	*mac32*; *zu135/nT1[qIs51]*	Adult
	*mac32*; *zu135*	Larval arrest
	*mac35*; *zu135/nT1[qIs51]*	Adult
	*mac35*; *zu135*	Larval arrest

### wdr-23(lf) and skn-1(gf) interact with bli-3(lf) differentially to affect animal survival in high concentration of NaI

To further understand the interactions of *bli-3* with *skn-1* and *wdr-23*, we chose two independent alleles of each of the three genes and generated double mutants. We examined the survival of single or double mutants in 10 mM or 50 mM NaI. Higher concentrations of iodide might cause more severe oxidative stress, which can be used for detecting additive or synergistic genetic interactions (Table 4).

**Table 4:** The survival of single and double mutants in 10 or 50 mM NaI

Genotype	Alleles	Survival in 10 mM NaI	Survival in 50 mM NaI
*bli-3(lf)*	*e767*	Yes	No
	*mac40*	Yes	No
*skn-1(gf)*	*lax120*	Yes	No
	*mac53*	Yes	No
*wdr-23(lf)*	*mac32*	Yes	No
	*mac35*	Yes	No
*bli-3(lf)*; *skn-1(gf)*	*e767*; *mac53*	Yes	No
	*e767*; *lax120*	Yes	No
	*mac40*; *mac53*	Yes	Yes
	*mac40*; *lax120*	Yes	Yes
*bli-3(lf) wdr-23(lf)* First group	*e767 mac32*	Yes	No
	*e767 mac35*	Yes	No
	*mac40 mac32*	Yes	No
	*mac40 mac35*	Yes	No
*bli-3(lf) wdr-23(lf)* Second group	*mac40 mac36*	Yes	No
	*mac40 mac42*	No	No
	*mac40 mac44*	Yes	No
	*mac40 mac51*	Yes	No

We found that all single and double mutants can survive in 10 mM NaI (Table 4), suggesting that at this concentration iodide does not generate a lethal oxidative stress. We next tested 50 mM NaI, in which all single mutants failed to survive (Table 4). Interestingly, *bli-3(lf)*; *skn-1(gf)* double mutants exhibited split phenotypes: the two *bli-3(e767lf)*; *skn-1(gf)* double mutants failed to survive in 50 mM NaI, while the two *bli-3(mac40lf)*; *skn-1(gf)* double mutants could survive. Different from *bli-3(lf)*; *skn-1(gf)*, all four *bli-3(lf) wdr-23(lf)* double mutants we initially generated failed to survive (Table 4, First group). Therefore, *skn-1(gf)* and *wdr-23(lf)* can interact with *bli-3(mac40lf)* differentially.

To examine whether other *wdr-23* alleles might interact with *bli-3(mac40lf)* in a similar manner, we generated new *bli-3(mac40lf) wdr-23(lf)* double mutants that include four more *wdr-23* missense or nonsense mutations (Table S2). All these *bli-3(mac40lf) wdr-23(lf)* double mutants also failed to survive in 50 mM NaI (Table 4, Second group).

In all double mutants, only *bli-3(mac40lf) wdr-23(mac42lf)* could not survive in 10 mM NaI. We speculate that an unknown defect(s) in the double mutants that is not derived from oxidative stress contributes to the inviability, since single mutants of either mutation could survive in 10 mM NaI.

### skn-1(gf) and wdr-23(lf) interact with bli-3 differentially to affect the cuticle integrity

A critical function of the BLI-3 dual oxidase is to catalyze the crosslinking of tyrosyl residues of the cuticle collagens. The process involves the BLI-3 NADPH oxidase domain, the BLI-3 peroxidase domain and the peroxidase MLT-7 (Edens *et al.* 2001; Meitzler and Ortiz De Montellano 2009; Thein *et al.* 2009; Meitzler *et al.* 2010; Moribe and Mekada 2013). To examine whether *skn-1* or *wdr-23* interacts with *bli-3* to affect cuticle formation, we tested the cuticle integrity of mutants by staining with the fluorescent nuclear dye Hoechst 33258 (Thein *et al.* 2009; Xu *et al.* 2014).

**Figure 3:**
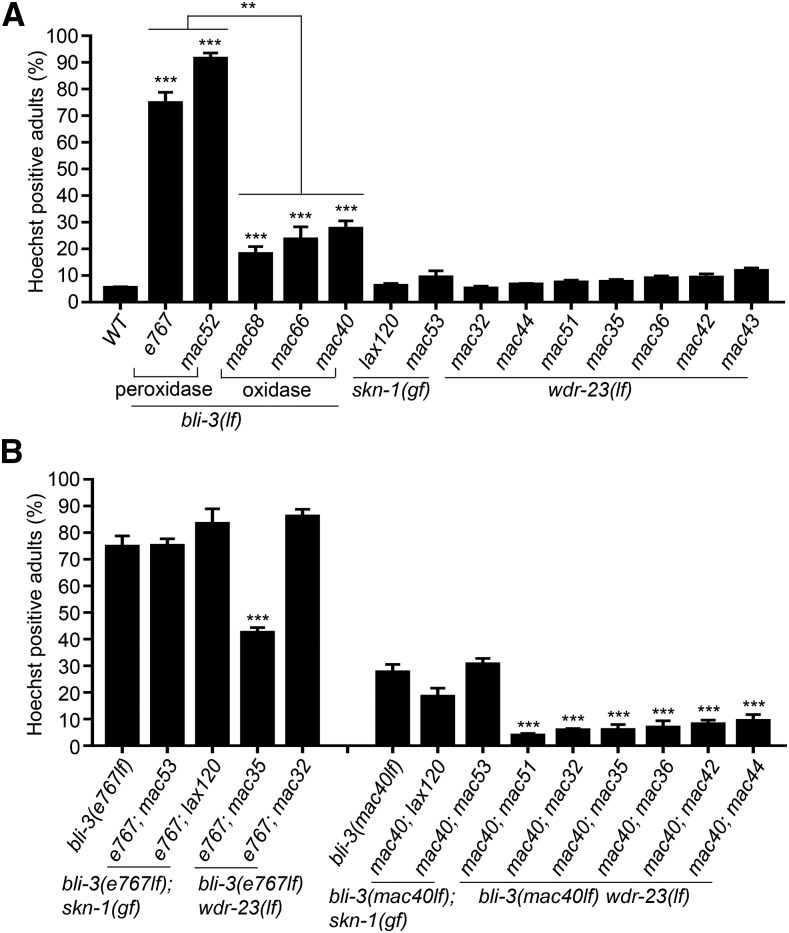
Cuticle integrity of single and double mutants based on Hoechst 33258 staining. (A) Percentage of Hoechst-positive single mutants. (B) Percentage of Hoechst-positive double mutants compared to single mutants. Results are from four biological replicates (n = 100 for each). Comparisons were made with WT or between genotypes. Error bar: Mean ± SE. Statistics: Bonferroni test with one-way ANOVA. *: *P* < 0.05; **: *P* < 0.01; ***: *P* < 0.001.

An examination of *bli-3* single mutants suggests that the peroxidase domain and the oxidase domain affect cuticle integrity differentially: the peroxidase domain mutations (*mac52* and *e767*) resulted in cuticles more defective than the oxidase domain mutations did (*mac68*, *mac66*, *mac40*) (Figure 3A). None of the *skn-1(gf)* or *wdr-23(lf)* single mutants has apparently defective cuticles (Figure 3A), suggesting that these two genes are not directly involved in cuticle formation.

An examination of double mutants suggests that *skn-1(gf)* mutations do not apparently alter the cuticle defects caused by mutations affecting either the peroxidase domain or oxidase domain of BLI-3 (Figure 3B). Surprisingly, *wdr-23(lf)* mutations affect the cuticles in a *bli-3* allele-specific manner: they uniformly and strongly suppress the cuticle defects of the *bli-3(mac40lf)* (oxidase domain) mutants but not that of *bli-3(e767lf)* (peroxidase domain) mutants (Figure 3B).

### skn-1(gf) and wdr-23(lf) similarly affect the expression of most, but not all target genes

To understand what downstream genes of *skn-1* and *wdr-23* might be involved in promoting animal survival in excess iodide, we performed RNA-Seq on synchronized wild-type, *skn-1(mac53gf)* and *wdr-23(mac35lf)* larvae grown with or without excess iodide and analyzed their transcriptomes.

**Figure 4:**
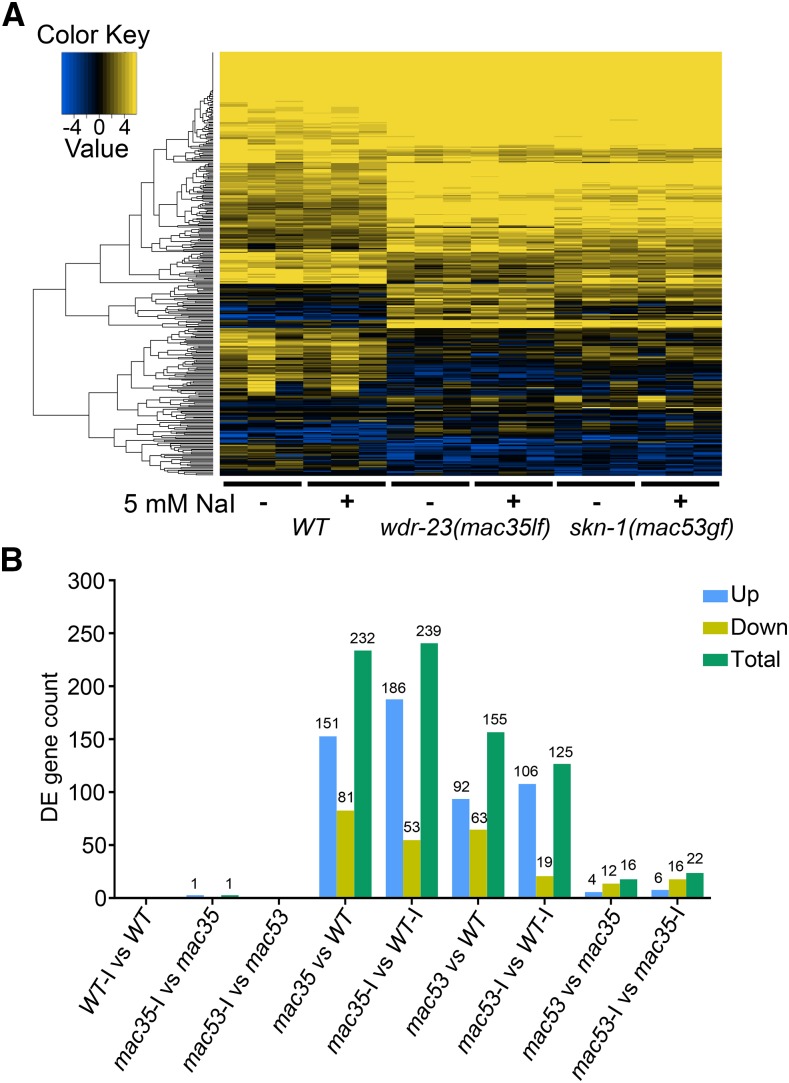
Transcriptome analyses of DEGs in wild-type and mutant animals grown with or without excess iodide. (A) Heat map of differentially expressed genes (DEGs). Results are from three biological replicates. (B) Numbers of DEGs from pairwise comparisons. Iodide treatment is indicated as “-I”.

We found that animals of the same genotype exhibit largely similar gene expression profiles with or without excess iodide, as shown in the gene expression heat map (Figure 4A) and the numbers of differentially expressed genes (DEGs) (Figure 4B). The gene expression profiles of *skn-1(mac53gf)* and *wdr-23(mac35lf)* mutants are apparently different from that of wild type, while a subtle but visible difference is also seen between them (Figure 4A). Fewer genes are altered in *skn-1(mac53gf)* mutants compared to *wdr-23(mac35lf)* mutants (Figure 4B). Direct comparison of *skn-1(mac53gf)* and *wdr-23(mac35lf)* identified 16 (without iodide) and 22 (with iodide) DEGs (Figure 4B).

Gene ontology (GO) analyses revealed that many of the DEGs in *skn-1* and/or *wdr-23* mutants belong to signaling pathways that regulate metabolism and defense response, with glutathione metabolic process being the top pathway in each mutant (Fig. S2 and S3). Cellular process, metabolic process and single-organism process are the top three GO subterms that contain the highest percentage of DEGs in each mutant (Fig. S4).

A few GO subterms are differentially affected between *wdr-23(mac35lf)* and *skn-1(mac53gf)* mutants. For example, innate immune response is one biological process that is significantly affected by *wdr-23(mac35lf)* but not by *skn-1(mac53gf)* (Fig. S3A). Glutathione and alkyl or aryl transferase activities are the two molecular functions that are significantly different between *skn-1(mac53gf)* and *wdr-23(mac35lf)* mutants (Fig. S3B).

### skn-1(gf) and wdr-23(lf) can affect gene expression differentially

We compared the DEGs in each mutant (Fig. S5). The list of shared genes indicates that *gst* and *ugt* genes are the most represented antioxidant gene categories among the up-regulated groups (Result S1). Interestingly, *wdr-23* is significantly up-regulated in each mutant, suggesting an autoregulation of *wdr-23* expression by the SKN-1/WDR-23 pathway (Result S1).

**Figure 5:**
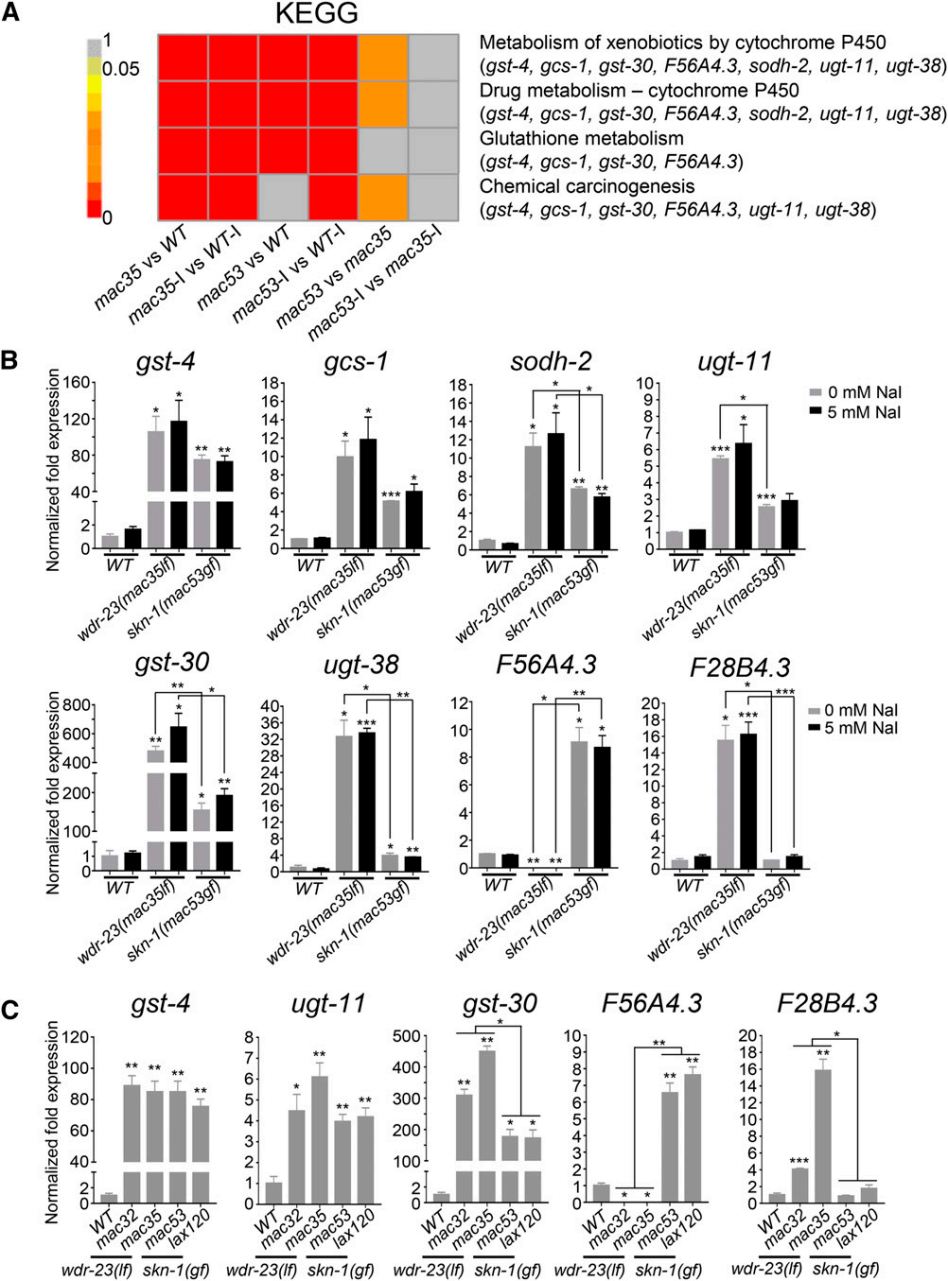
qRT-PCR verification of altered gene expression. (A) Top four pathways and seven candidate genes that are altered in both *wdr-23(lf)* and *skn-1(gf)* mutants based on KEGG analysis. Among the candidate genes, *gst-30,F56A4.3* and *ugt-38* are also differentially expressed between *skn-1(mac53gf)* and *wdr-23(mac35lf)* based on RNA-Seq. (B) qRT-PCR examination of the eight *skn-1/wdr-23* target genes in different genotypes treated with or without excess iodide. (C) qRT-PCR examination of the expression of five *skn-1/wdr-23* target genes in independent mutants of *skn-1* or *wdr-23*. Results are from three biological replicates. Reference gene: *tba-1*. Comparisons were made with wild type or between genotypes. Error bars: Mean ± SE. Statistics: two-tailed unpaired Student’s *t*-test. *: *P* < 0.05; **: *P* < 0.01; ***: *P* < 0.001.

Compared to wild type, the top four KEGG pathways affected by *skn-1(mac53gf)* and *wdr-23(mac35lf)* include glutathione metabolism, cytochrome P450-related drug and xenobiotics metabolisms, and chemical carcinogenesis (Figure 5A). We selected seven genes with the highest differential expression in these four comparisons to verify their expression by qRT-PCR. The increased expression of the first six genes (*gst-4*, *gcs-1*, *sodh-2*, *ugt-11*, *gst-30* and *ugt-38*) is verified in both *skn-1(mac53gf)* and *wdr-23(mac35lf)* mutants (Figure 5B). Compared to *wdr-23(mac35lf)*, the expression of *sodh-2*, *ugt-11*, *gst-30* and *ugt-38* is less increased in *skn-1(mac53gf)* mutants (Figure 5B).

The seventh gene, *F56A4.3*, exhibits a completely different expression: its expression is significantly increased in *skn-1(mac53gf)* mutants but essentially abolished in *wdr-23(mac35lf)* mutants (Figure 5B). *F56A4.3* encodes a glutathione S-transferase with unknown function (www.wormbase.org).

We examined whether the expression of other genes is also differentially affected by *skn-1(gf)* and *wdr-23(lf)*. An examination of DEGs between *skn-1(gf)* and *wdr-23(lf)* mutants (Result S2) identified *F28B4.3*: qRT-PCR indicates that its expression is significantly increased in *wdr-23(mac35lf)* but remains unaltered in *skn-1(mac53gf)* mutants (Figure 5B).

To determine whether the differential effects on gene expression by *skn-1(mac53gf)* and *wdr-23(mac35lf)* are allele-specific, we examined the expression of a subset of these genes in *skn-1(lax120gf)* and *wdr-23(mac32lf)* mutants (Figure 5C). The expression of these genes exhibits similar patterns in the two independent mutants of *skn-1* or *wdr-23* (Figure 5C), suggesting that *skn-1(gf)* and *wdr-23(lf)* can affect the expression of a subset of genes, *e.g.*, *F56A4.3* and *F28B4.3*, by a mechanism different from the one used by their shared *gst* and *ugt* target genes.

## Discussion

In this study, we isolated multiple mutations affecting *C. elegans skn-1*, *wdr-23* and the *bli-3/tsp-15/doxa-1* complex by screening for surviving mutants in excess iodide. We suggest that WDR-23 and SKN-1 interact with the BLI-3/TSP-15/DOXA-1 complex to regulate animal’s response to oxidative stress. We also suggest that WDR-23 loss of function can affect BLI-3 activity and some gene expression independent of SKN-1 activation.

### SKN-1/WDR-23 and BLI-3/TSP-15/DOXA-1 affect C. elegans survival in excess iodide by a conserved mechanism

SKN-1 and BLI-3 are involved in *C. elegans* response to pathogens (Hoeven *et al.* 2011; Tang and Pang 2016), oxidative stress (Ewald *et al.* 2017), manganese toxicity (Benedetto *et al.* 2010) and ROS-related lifespan extension (Sasakura *et al.* 2017). The BLI-3/TSP-15/DOXA-1 complex is also required for the formation of *C. elegans* cuticles by generating H_2_O_2_, which is utilized by the BLI-3 peroxidase domain (Edens *et al.* 2001) and the peroxidase MLT-7 for crosslinking cuticle proteins (Thein *et al.* 2009). The concurrent involvement of SKN-1 and BLI-3 in multiple cellular processes suggests functional crosstalk between these two molecules and/or the pathways. It is unclear whether and how the oxidase and peroxidase activities of BLI-3, the activation of SKN-1, and the ROS production are coordinated *in vivo*.

Our screening for recessive and dominant mutations that can promote animal survival in excess iodide identified lf mutations in the *bli-3/tsp-15/doxa-1* complex and *wdr-23* and gf mutations in *skn-1*. It is plausible that the reduced ROS generation in *bli-3/tsp-15/doxa-1* lf mutants and the activation of antioxidant gene expression in *wdr-23(lf)* or *skn-1(gf)* mutants would attenuate the oxidative stress caused by excess iodide, a strong inducer of ROS in *C. elegans* and mammals (Many *et al.* 1992; Golstein and Dumont 1996; Corvilain *et al.* 2000; Vitale *et al.* 2000; Yao *et al.* 2012; Serrano-Nascimento *et al.* 2014; Xu *et al.* 2014). Consistent with this hypothesis, we found that the antioxidants ascorbic acid (vitamin C) and N-acetylcysteine (NAC) can antagonize the toxic effect of excess iodide (Table S6).

Recent studies found that excess iodide could increase Nrf2 expression in rat thyroid (Wang *et al.* 2017) and activate the Nrf2 pathway in human skin cells (Ben-Yehuda Greenwald *et al.* 2017). Therefore, it is a conserved mechanism that the BLI-3/TSP-15/DOXA-1 dual oxidase complex and the Nrf2/SKN-1 pathway are both involved in the response to oxidative stress induced by excess iodide.

### SKN-1/WDR-23 and BLI-3/TSP-15/DOXA-1 likely function in the hypodermis to affect C. elegans survival in excess iodide

In *C. elegans*, *skn-1* and *wdr-23* are expressed in intestine, hypodermis and other tissues (An and Blackwell 2003; Hasegawa *et al.* 2008; Choe *et al.* 2009; Hasegawa and Miwa 2010; Paek *et al.* 2012; Wu *et al.* 2016) and the anti-stress functions of SKN-1C have been primarily associated with its intestinal expression (Blackwell *et al.* 2015). *bli-3* is also expressed in intestine and hypodermis (Edens *et al.* 2001; Van Der Hoeven *et al.* 2015), while *tsp-15* appears to be expressed primarily in hypodermis (Moribe *et al.* 2004). We could phenocopy the survival-promoting effect of *skn-1c(gf)* mutation or rescue that of *tsp-15(lf)* mutation by hypodermis-specific expression of *skn-1c(gf)* or *tsp-15(wt)* transgenes, respectively (Tables 2 and S3), suggesting that SKN-1/WDR-23 and BLI-3/TSP-15/DOXA-1 function in the hypodermis to affect the oxidative stress effect of excess iodide.

How *C. elegans* takes in iodide is unknown. In mammals, iodide uptake is mediated by Na(+)/I(-) symporter (NIS), an integral plasma membrane glycoprotein expressed in multiple tissues including thyroid, the lacrimal sac and nasolacrimal duct, salivary glands, choroid plexus, stomach, intestine, lactating breast, kidney, placenta and ovary (Ravera *et al.* 2017). We previously found that RNAi targeting two *C. elegans* genes similar to *NIS* did not apparently affect animal survival in excess iodide (Xu *et al.* 2014). It is possible that iodide is absorbed by the intestine and then transported to the hypodermis in *C. elegans*. Alternatively, iodide might gain access to the hypodermis directly via microscopic openings on the cuticle. The detailed mechanism remains to be understood.

### SKN-1C is the primary SKN-1 isoform responsible for promoting animal survival in excess iodide

The SKN-1C isoform normally resides in the cytoplasm and enters the nucleus in response to stress signals (An and Blackwell 2003; Blackwell *et al.* 2015). The SKN-1A isoform might be associated with mitochondria (Paek *et al.* 2012) to mediate starvation response. It is also associated with ER (Lehrbach and Ruvkun 2016) to respond to proteasome dysfunction signals. The *skn-1* gf mutations we identified affect both A and C isoforms (Fig. S1). Our transgene experiments suggest that SKN-1C is the major isoform that promotes animal survival, while SKN-1A might play a minor role. Hypodermic overexpression of SKN-1C(gf) might be highly toxic, which explains why we failed to obtain any stable transgenic lines using the *skn-1c* promoter or the hypodermis-specific *dpy-7* promoter and suggests that a highly regulated SKN-1C activity in hypodermis is essential for development and survival.

In WormBase (www.wormbase.org), five WDR-23 isoforms are annotated. The mutations we isolated affect all *wdr-23* isoforms (Figure 1D and Fig. S6). Previous studies found that the WDR-23A isoform is associated with outer mitochondrial membranes, while the WDR-23B isoform is localized exclusively in the nucleus (Staab *et al.* 2013; Staab *et al.* 2014). We found that *wdr-23a* and *wdr-23b* transgenes can strongly rescue the phenotype of *wdr-23(lf)* mutants with a similar efficiency (Table S3), which is consistent with the previous finding that a functional difference of the two WDR-23 isoforms was not detected in transgenic experiments (Staab *et al.* 2013).

### The peroxidase domain and oxidase domain of BLI-3 are functionally divergent

BLI-3 is the only functional dual oxidase in *C. elegans* that contains an N-terminal peroxidase domain and a C-terminal oxidase domain (Edens *et al.* 2001; Donko *et al.* 2005; Bedard *et al.* 2007). It is also the only NADPH oxidase in *C. elegans* (Bedard *et al.* 2007). Studies of missense mutations affecting the peroxidase domain or the oxidase domain of BLI-3 found that peroxidase mutations do not or only weakly affect infection-induced H_2_O_2_ production, while an oxidase mutation (Moribe *et al.* 2012) has an apparently stronger effect (Chavez *et al.* 2009; Van Der Hoeven *et al.* 2015). The oxidase domain mutation could also reduce the lifespan and make *C. elegans* more susceptible to pathogens, while the peroxidase mutations do not or only weakly do so (Chavez *et al.* 2009; Van Der Hoeven *et al.* 2015).

In our study, BLI-3 peroxidase domain mutations impair the cuticle integrity more severely than oxidase mutations do (Figure 3A), suggesting a functional difference of these two domains that is consistent with previous findings (Chavez *et al.* 2009; Van Der Hoeven *et al.* 2015). We previously found that ROS overproduction caused by excess iodide in *C. elegans* can be partially suppressed by both the *bli-3(e767lf)* (peroxidase) and *bli-3(mac40lf)* (oxidase) mutations (Xu *et al.* 2014), suggesting that BLI-3 peroxidase domain mutations also impact the oxidase domain. Therefore, the peroxidase domain [that consumes ROS to crosslink tyrosyl residues of collagens] and the oxidase domain [that generates ROS] likely interact and also function differentially to affect cuticle formation, ROS generation and the response to oxidative stress or pathogens.

### WDR-23 might carry out a function independent of SKN-1 activation

*wdr-23(lf)* and *skn-1(gf)* interact with *bli-3(lf)* differentially. First, only *bli-3(mac40lf)*; *skn-1(gf)* double mutants could survive in 50 mM NaI, while other double mutants, including all *bli-3(lf) wdr-23(lf)* and *bli-3(e767lf)*; *skn-1(gf)* mutants, could not (Table 4). Second, *wdr-23(lf)* mutations could strongly suppress the cuticle defects of only *bli-3(mac40lf)* mutants but not that of *bli-3(e767lf)* mutants, while *skn-1(gf)* mutations do not suppress the cuticle defects of either *bli-3(lf)* mutants (Figure 3). These findings imply that WDR-23 might suppress BLI-3 activity. Since WDR-23 inhibits SKN-1 activation by promoting proteasome-mediated SKN-1 degradation (Choe *et al.* 2009), it is plausible that WDR-23 might inhibit BLI-3 or a protein required for BLI-3 activity by a similar mechanism. Future studies are warranted to test this hypothesis.

Our transcriptome analyses suggest that excess iodide does not apparently alter gene expression in wild-type or mutant *C. elegans* (Figure 4). The survival-promoting effect of *skn-1(gf)* and *wdr-23(lf)* mutations likely involve the activation of antioxidant gene expression (Fig. S2, S3, S4 and Result S1), similar to what previous studies have found (Blackwell *et al.* 2015). However, *skn-1(gf)* and *wdr-23(lf)* can differentially affect the expression of a subset of genes. For example, *wdr-23(lf)* causes higher expression of four genes that are up-regulated in both *wdr-23(lf)* and *skn-1(gf)* mutants (Figure 5B and 5C, *sodh-2*, *ugt-11*, *gst-30*, *ugt-38*). The expression of *F56A4.3* is abolished in *wdr-23(lf)* mutants but significantly increased in *skn-1(gf)* mutants (Figure 5B and 5C), while the expression of *F28B4.3* is significantly increased in *wdr-23(lf)* mutants but unaltered in *skn-1(gf)* mutants (Figure 5B and 5C). These differences are not allele-specific for *wdr-23* or *skn-1*, suggesting a potentially novel mechanism underlying these effects of WDR-23 loss of function and SKN-1 gain of function. The biological significance of this differentiation remains unclear.

**Figure 6:**
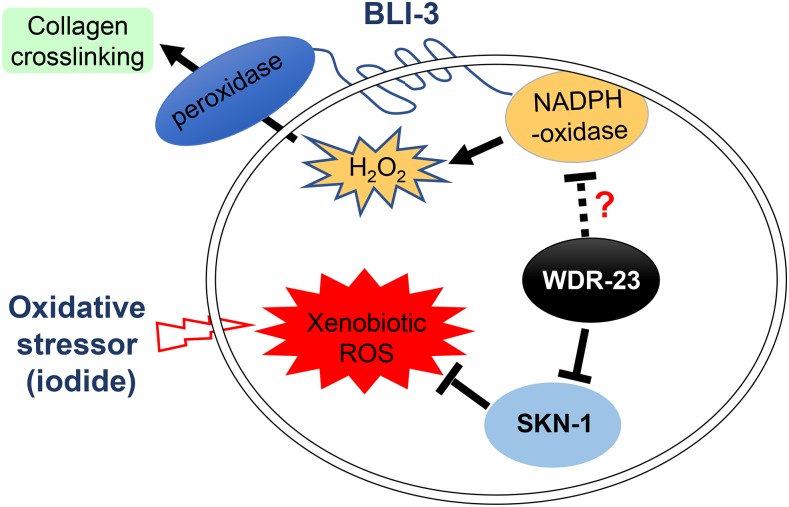
A working model describing the known and potential interactions among SKN-1, WDR-23 and BLI-3. In this model, BLI-3 contributes to oxidative stress by generating H_2_O_2_ used for crosslinking cuticle collagens. Besides inhibiting the activation of SKN-1, WDR-23 might also suppress BLI-3 by an unknown mechanism.

We generated a working model to describe the known and potential interactions among SKN-1, WDR-23 and BLI-3 (Figure 6). In this model, the H_2_O_2_ generated by BLI-3 for crosslinking cuticle collagens also contributes to the oxidative stress caused by xenobiotic stressors such as iodide. Besides its canonical role as a SKN-1 negative regulator, WDR-23 might also suppress BLI-3 activity, a possibility supported by the genetic interaction between *wdr-23(lf)* and *bli-3(lf)* (Figure 3 and Table 4).

In summary, we found that the oxidative stress triggered by excess iodide can be suppressed by defects in the BLI-3/TSP-15/DOXA-1 complex or the activation of SKN-1 either by *skn-1(gf)* or *wdr-23(lf)* mutations. We provide further genetic and molecular evidence supporting the consensus that WDR-23 can function as a negative regulator of SKN-1 in activating antioxidant gene expression and also suggest that WDR-23 could interact with BLI-3 and affect some gene expression by a mechanism(s) different from the prior one. Our findings should facilitate the understanding of animal’s response to oxidative stress. Future studies are warranted for elucidating the underlying molecular mechanism.
